# Identification of Potential Drug Targets in Cancer Signaling Pathways using Stochastic Logical Models

**DOI:** 10.1038/srep23078

**Published:** 2016-03-18

**Authors:** Peican Zhu, Hamidreza Montazeri Aliabadi, Hasan Uludağ, Jie Han

**Affiliations:** 1 Department of Electrical and Computer Engineering, University of Alberta, Edmonton, AB T6G 1H9, Canada; 2 School of Pharmacy, Chapman University, Irvine, CA 92618, USA; 3 Department of Chemical and Material Engineering, University of Alberta, Edmonton, AB T6G 1H9, Canada

## Abstract

The investigation of vulnerable components in a signaling pathway can contribute to development of drug therapy addressing aberrations in that pathway. Here, an original signaling pathway is derived from the published literature on breast cancer models. New stochastic logical models are then developed to analyze the vulnerability of the components in multiple signalling sub-pathways involved in this signaling cascade. The computational results are consistent with the experimental results, where the selected proteins were silenced using specific siRNAs and the viability of the cells were analyzed 72 hours after silencing. The genes elF4E and NFkB are found to have nearly no effect on the relative cell viability and the genes JAK2, Stat3, S6K, JUN, FOS, Myc, and Mcl1 are effective candidates to influence the relative cell growth. The vulnerabilities of some targets such as Myc and S6K are found to vary significantly depending on the weights of the sub-pathways; this will be indicative of the chosen target to require customization for therapy. When these targets are utilized, the response of breast cancers from different patients will be highly variable because of the known heterogeneities in signaling pathways among the patients. The targets whose vulnerabilities are invariably high might be more universally acceptable targets.

Biological functions are implemented through the interactions among genes, proteins and other intracellular molecules. The chains of interactions, i.e., pathways that mediate the signals inside cells, are being actively investigated to better understand normal and defected processes. Cancer is known as a heterogeneous disease. In fact, extensive genetic diversity has been revealed not only between different types of cancer, but also within a single tumor (as diversity in the expression of protein biomarkers)^1^. This intratumor heterogeneity might be a consequence of genetic changes, environmental factors, and/or variations in cell properties^2^, and could be a major obstacle in cancer treatment due to a wide range of responsivity to any specific anticancer agent. Breast cancer, specifically, demonstrates significant heterogeneity from onset^3^. Therefore, study of multiple intracellular pathways involved in cancer cells with enhanced survival and proliferation could provide variable information about the role of each component, cross-talk between the pathways, and ultimately, the efficacy of targeting each protein in controlling the tumor growth.

Pathway biology aims to understand the cause-effect relationships among genes and such a system-level study is intended to integrate the information in published investigations to provide further understanding of pathways^4^,^5^. Various approaches have been proposed in order to provide some insight in pathway analysis, including logical models^6^,^7^, continuous models using differential equations^8^,^9^ and other models^10^. A logic model is used as an informative and effective approach to modelling the biological pathways^11^,^12^. In particular, Boolean networks (BNs) have been widely used to qualitatively model the interactions among genes^6^. The activation and inhibition relationships are modeled by digital logic where the activation levels of gene nodes are indicated by ON/OFF states. Probabilistic and Stochastic Boolean networks (PBNs and SBNs) have been used to efficiently simulate gene networks^13^,^14^,^15^,^16^.

Various studies have been carried out to estimate the robustness or vulnerability of a system^17^,^18^,^19^. In a biological network, dysfunction of a gene node in a pathway may result in the transition from a normal state to a defective one. A better understanding of the importance of nodes has provided insights for identification of potential new drug targets^20^,^21^,^22^,^23^.

Here, the vulnerability of gene nodes in a breast cancer signaling pathway constructed from the literature is investigated. The vulnerable nodes are intended to serve as viable drug targets where a therapeutic benefit is more likely to be obtained. We constructed and modeled critical signaling pathways, and undertook inhibition of several nodes in order to assess the predictions of the constructed model. New stochastic Boolean network models were developed and utilized to provide a quantitative evaluation of the vulnerability of each gene node in the constructed pathway.

## Methods

### Breast cancer pathway derivation

**Figure 1:**
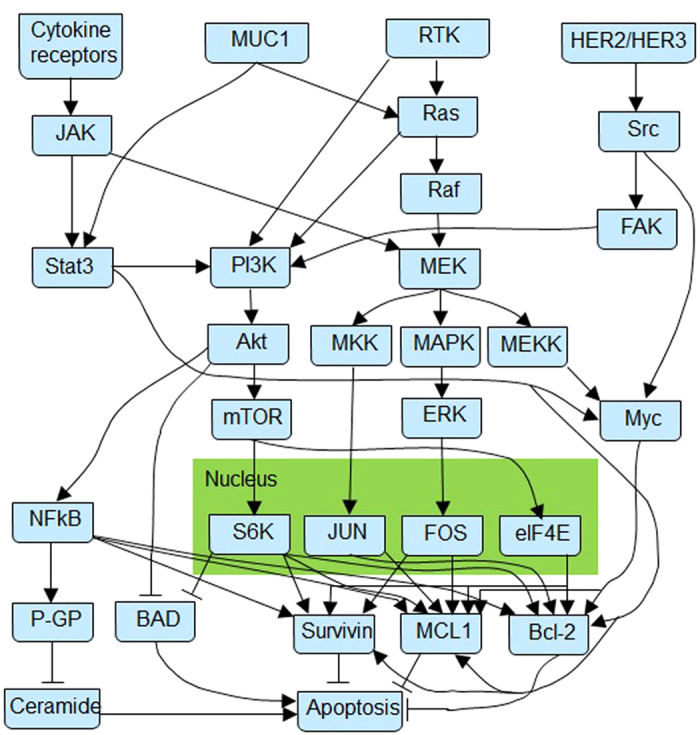
Breast cancer signaling pathway derived from the literature. An arrow indicates the positive regulatory interaction or activation between two genes, while a blunt arrow represents an inhibition relationship.

The structure of the signaling pathway was derived from the breast cancer models published in the literature. The constructed pathway has four membrane components listed as cytokine receptors, Mucin-1 (MUC1), receptors of tyrosine kinase (RTKs) and human epidermal growth factor receptor 2/3 (HER2/HER3). The family of cytokine receptors consist of 40 different membrane receptors for interleukins, interferons, tumor necrosis factors (TNF), and chemokines^24^. Engagement of these receptors activates the associated JAK2s^25^, which in turn triggers JAK2-STAT signalling pathway in different cancer cells. MUC1 is a glycosylated mucin expressed on the apical surface of the ducts and glands of simple secretory epithelial tissues (e.g., mammary gland, and gastrointestinal and respiratory tracts^26^). Over-expression of MUC1 is reported in many carcinomas, and specifically in breast cancer^27^, and is correlated with higher metastasis risk and poor survival rate^28^. MUC1 protein interacts with several cytoplasmic proteins involved in neoplastic transformation and enhanced cell proliferation, including Ras-MEK-ERK2 signaling pathway^29^, downstream proteins to the epidermal growth factor receptor (EGFR^30^), and Stat3 (via Src signalling pathway^31^). RTKs are a subset of the cell membrane growth factor receptors with tyrosine-kinase activity that is triggered via ligand association, and with an important role in oncogenesis^32^. Direct connection of RTK activation with both Ras-Raf and PI3K-Akt pathways has been established in several studies. HER2/HER3, on the other hand, are members of the epidermal growth factor family of transmembrane receptors (Erbb family) that trigger signalling pathways regulating cell proliferation, among an array of cellular functions^33^. Based on the above information, a breast cancer signaling pathway was constructed, as shown in Fig. 1.

### Sub-pathway division

**Figure 2:**
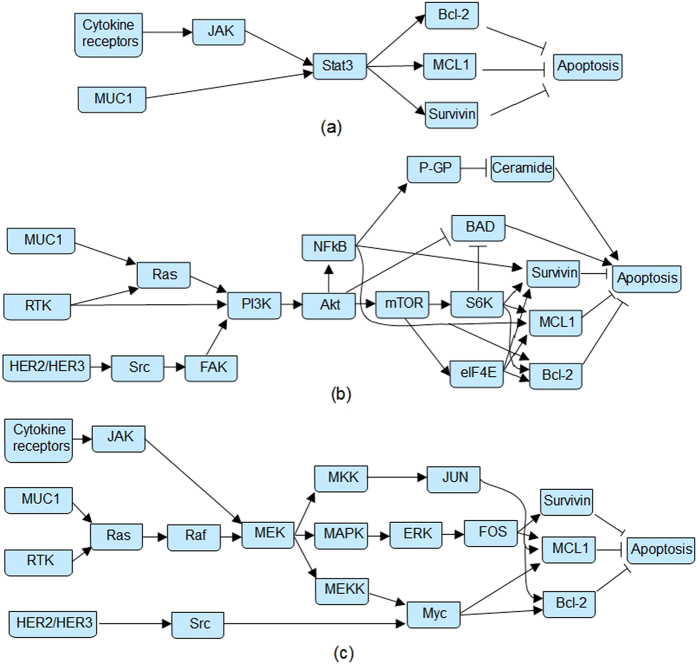
The three sub-pathway divisions. (**a**) sub-pathway 1 (Stat3); (**b**) sub-pathway 2 (PI3K); (**c**) sub-pathway 3 (Ras-Raf). An arrow indicates a positive regulatory interaction or activation between two genes, while a blunt arrow represents an inhibition relationship.

While the generated signaling model is a broadly acting pathway in breast cancer cells, it is known that not all components of the constructed pathway shown in Fig. 1 are necessarily over-activated (or over-expressed) in all breast cancer cells. For example, HER2 overexpression is reported in 18–25% of human breast cancer cases^34^ and mutations in genes that constitute the PI3K pathway occur in 70% of breast cancers^35^. In order to account for the dominance of particular components in different kinds of cells, the derived pathway in Fig. 1 was divided into three axes (Fig. 2) based on the central roles of three distinct signaling pathways with different weights assigned to each axis: (a) JAK2-STAT axis, which is mainly triggered by the JAK2 kinase associated with cytokine receptors; (b) PI3K-Akt axis, which is mainly triggered by the Receptor Tyrosine Kinases, activates multiple proteins responsible for cell proliferation via kinase PI3K and serine/threonine kinase Akt; and (c) Ras-Raf axis, which activates MEK-ERK pathway and downstream transcription factors via the GTPase Ras and serine/threonine kinases Raf. By assigning a higher percentage to each sub-pathway, we recognize the more prominent role of that sub-pathway in signaling. Obviously, each axis is not independent and there are inevitable interactions or crosstalk among them. The interactions are modeled in two ways. The first is through the sharing of common signaling molecules and/or paths in more than one sub-pathways. Examples of this include the survivin-apoptosis interaction in all three sub-pathways, the cytokine-JAK2 interaction in sub-pathways 1 and 3, the MUC1/GFs and Ras interaction in sub-pathways 2 and 3. The effect of these crosstalks is then evaluated by assigning different weights to each of the sub-pathways. In this way, the importance of each sub-pathway can be independently controlled on particular breast cancer cells. To have an unbiased evaluation, 16 strategies that assigned different weights to each sub-pathway are selected to uniformly cover a wide range of combinations of the three subpathways (i.e., they represent the spectrum of possibilities). These 16 strategies are then simulated and the results are compared to experimental results to find out which strategies are more realistic in a biological context.

### Experimental gene silencing and analysis

Short interfering RNA (siRNA) mediated gene silencing was employed as a therapeutic approach to determine sensitivity of cells to selected targets. siRNA therapy, due to its specificity and potency, is currently explored for therapy of various cancers, including breast cancers. By inhibiting specific nodes in the constructed signaling pathway, we sought to experimentally assess the importance of the selected nodes on cell viability. The details of our experimental gene silencing procedures were extensively reported previously^36^. Briefly, wild-type MDA-MB-435 cancer cells (American Cell Type Collection, ATCC; Manassas, VA) were seeded in 24-well plates at ~20% confluency (~1.5 × 10^5^ cells/mL) and treated with desired siRNA complexes after 24 hours. The MDA-MB-435 cell line is believed to represent a poorly differentiated, aggressive breast tumor line, with expression of both epithelial and melanocytic markers^37^. The siRNA complexes were prepared by incubating commercially available siRNAs (see Table S1 for the sequence and source of siRNAs) with a polymer solution (linoleic acid-substituted 2 kDa branched polyethylenimine, PEI-LA; 2.0 LA/PEI)^36^. The complexes were added to the wells to give siRNA concentrations of 9, 18, and 36 nM (in 0.5 mL tissue culture medium). The plates were incubated in a humidified atmosphere of 95/5% air/*CO*_2_ at 37 °C for 72 hours before evaluation of cell viabilities. After 72 hours incubation with the complexes, the reduction in cell viabilities was measured by the MTT assay^36^. This time point was chosen based on previous studies, which indicated it to be optimal for assessment of treatment efficacies^36^. Cell viabilities were expressed as a percentage of untreated cells (taken as 100% viability). The cell viabilities obtained were used to obtain correlation with vulnerabilities obtained from simulations (below). Pearson’s correlation coefficient, *r*, was used for this purpose and the significance of the correlation was determined by Student t-distribution (*p* < 0.05).

In studies where the effect of specific siRNA treatment on mRNA levels were measured, the cells were exposed to the complexes at 18 nM and total RNA was isolated after 2 days as described in^36^. To synthesize the cDNA, 0.5 *μ*g total RNA was reverse transcribed by using random hexamer primer (See Table S2) and dNTP mix, and heated at 65 °C for 5 min. Synthesis buffer (5×), DTT (0.1 M), and RNAout RNase inhibitor (1.8 U/*μ*L) were added and the solutions were incubated at 37 °C for 2 min. MMLV RT enzyme was added to the solutions and incubated at 25 °C for 10 min, 37 °C for 50 min, and 70 °C for 15 min for cDNA synthesis. For amplification, 100 ng of the synthesized cDNA was mixed with 10× ThermPol Buffer, dNTP mix (5 mM), forward and reverse primers (3 *μ*M each) reverse, and Taq polymerase (5 U/*μ*L). The housekeeping gene *β*-actin was also amplified as controls. Real-time PCR was performed on an ABI 7500 HT with human *β*-actin (Forward: 5′-CCA CCC CAC TTC TCT CTA AGG A-3′; Reverse: 5′-AAT TTA CAC GAA AGC AAT GCT ATC A-3′) as the endogenous housekeeping gene and specific primers for each target. All the primers were tested to assure equal efficiency (with a slope < 0.1 for the Δ*C*_*T*_ vs. cDNA dilution graph), and a template concentration of 10 ng/*μ*L was determined as the optimal concentration based on the standard curves. Analysis was performed by calculating Δ*C*_*T*_, ΔΔ*C*_*T*_ , and Relative Quantity (RQ) compared to the “no treatment” group.

### Stochastic Boolean networks

**Figure 3:**
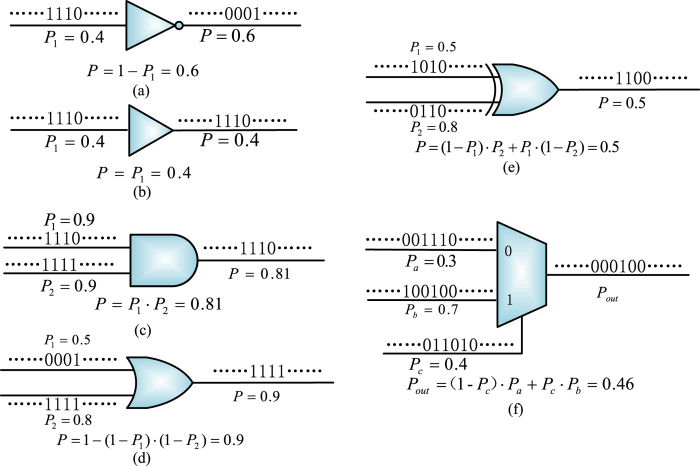
Logic gates for stochastic computation. (**a**) An inverter. (**b**) An identity gate. (**c**) An AND. (**d**) An OR. (**e**) An XOR. (**f**) A 2-to-1 multiplexer. A probabilistic computation is performed through stochastic logic operations by encoding signal probabilities into random binary bit sequences.

In stochastic computation, signal probabilities are directly encoded into random binary bit streams. Usually, a signal probability is encoded as a proportional number of bits being assigned to 1 in a binary stochastic sequence. Several stochastic processing elements are shown in Fig. 3, including an inverter, an identity gate, an AND, an OR, an XOR and a 2-to-1 multiplexer.

In stochastic logic, an inverter computes the complement of a probability. For the AND logic, if the two inputs are independent with probabilities *p*_1_ = 0.9 and *p*_2_ = 0.9, the output probability is expected to be *p*(1) = *p*_1_ · *p*_2_ = 0.9 × 0.9 = 0.81 (Fig. 3(c)). For a multiplexer (Fig. 3(f)), it takes one of the inputs as output according to the distributions of control bits (i.e., 0 and 1) in a stochastic sequence. The computational result obtained by stochastic logic is not deterministic but probabilistic due to inevitable stochastic fluctuations. Instead of using Bernoulli sequences, the stochastic fluctuation can be reduced through the use of non-Bernoulli sequences of random permutations of fixed numbers of 1’s and 0’s as initial inputs^38^. Signal correlations are efficiently handled in a stochastic network by the bit-wise dependencies in the random binary streams.

In a gene network or signaling pathway, the genetic interactions (either activation or inhibition) can be modeled by logic gates. As discussed previously, the dysfunction of certain gene nodes may transform a system from normal to a dysfunctional state. A targeting strategy is introduced to implement a ‘dysfunction’ of a gene node. The targeting process does not necessarily have to be deterministic so that a probabilistic drug delivery strategy is presented.

### Deterministic and probabilistic targeting

**Figure 4:**
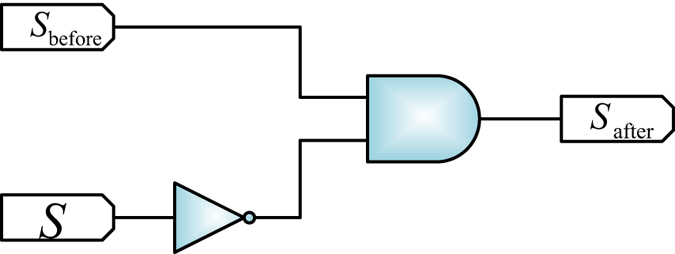
Drug intervention model for a binary gene node^39^. *S*_*before*_ and *S*_*after*_ denote the states of the target gene before and after gene silencing, respectively. *S* is a stochastic sequence encoding the functional probability of drug.

If drug intervention is adopted to suppress a gene node (referred to as a target) on the investigated pathway, the state of the target is driven to either 0 or 1, indicating suppressed or expressed target, respectively. Here, the effect of gene suppression under a particular type of drug (in our case, siRNA) is investigated to see the effect on the final output signal probability. Suppression of a gene was done by taking its state into zero. It is desirable for eradicating a cancerous cell by inducing cell death in order to block the deadly effects of uncontrolled cell growth in the long run. Drug intervention is modeled in Fig. 4^39^. If a drug intervention strategy is applied, i.e. Drug = 1, the state of the target gene becomes zero, as determined by the logic functions of the inverter (INV) and the AND gate, regardless of the initial expression level.

In practice, however, the outcome of drug intervention is usually not deterministic but it occurs with a certain probability. This process is referred to as a probabilistic drug delivery. The effect of probabilistic drug intervention can also be modeled by a combination of stochastic logic operations (as the model in Fig. 4, but with inputs given as stochastic sequences). Here, *S*_*before*_ and *S*_*after*_ denote the randomly permuted sequences of the target gene before and after drug delivery, respectively (encoding corresponding signal probabilities). The probabilistic drug intervention can be efficiently implemented by the stochastic structure. The evaluation accuracy improves with sequence length, but an accurate result can be obtained by using a reasonable sequence length.

### Stochastic node vulnerability analysis

**Figure 5:**
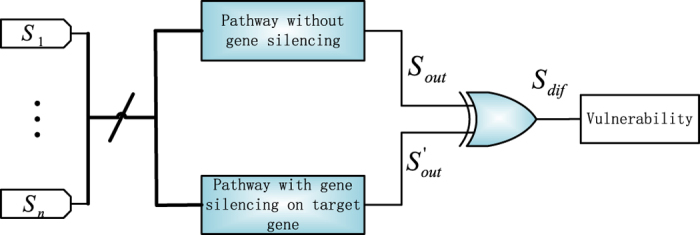
Stochastic model to compute the vulnerability of a target gene. *S*_*i*_ denotes the stochastic sequence of an input node, *i* ∈ {1, 2, 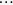, *n*}, where *n* is the total number of input genes in the investigated pathway. 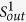 and 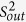 indicate the output sequences for the pathway without and with gene silencing, respectively.

Here, node vulnerability is defined as the probability that the output of the network, if that particular node is dysfunctional (for instance, silenced to zero), differs from that in a normal condition. Generally, dysfunction of each node could contribute to some extent to the different outputs of the entire pathway. The extent of contribution varies for different nodes because of the topology of the investigated pathway. In order to evaluate the node vulnerabilities, the stochastic Boolean network model is used to obtain the output signal probabilities. If a selected target gene is affected by the drug therapy, the deterministic or probabilistic drug intervention model in Fig. 4 can be applied to suppress the target gene. The model in Fig. 5 then computes the node vulnerability by comparing the output sequences of the pathways with and without gene silencing. In this model, each bit in a sequence can be considered as an experiment trial. If the result for such a trial with gene silencing is the same as the result without silencing, the silencing of the target gene has no effect on the expected output of the pathway. Otherwise, the target gene is considered to be vulnerable for changing the output of the pathway. By obtaining the statistics in the output sequence *S*_*dif*_ of the XOR gate in Fig. 5, the vulnerability value is determined by 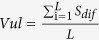, where *L* is the length of the stochastic sequences.

### Stochastic pathway construction

For a single interaction between two genes, “→” indicates an activation and is modeled by an Identity logic (i.e., the output is identical as the input), whereas “
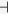
” indicates an inhibition and is modeled by an inverter or NOT logic. For multiple interactions with a single gene, the logical relationships are shown in Tables S3, S4 and S5 in the supplementary file. The complex relationships in the signaling pathway can be modeled by the AND or OR gate model^22^. If a node is controlled by multiple inputs and at least one of these inputs incur an inhibition, then the investigated node will be OFF as long as at least one of the inhibitors is ON. However, if a component does not have inhibitory inputs, then the investigated component will be ON as long as at least one of its inputs is ON. Furthermore, certain relationships are assumed to be primary according to obtained experimental results. For the three sub-pathways in Fig. 2(a–c), a stochastic model can be constructed for each sub-pathway. The input nodes are assumed to be with the same signal probability of 0.5; the output signal probability is obtained as the percentage of cell death.

**Figure 6:**
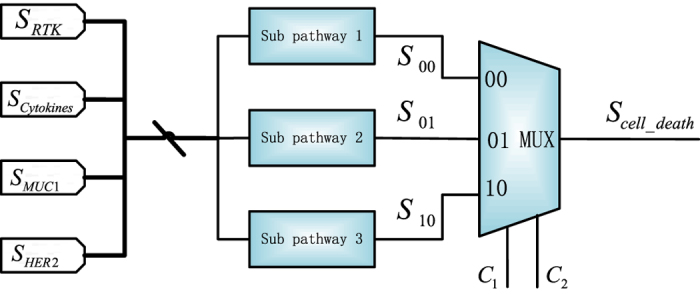
Stochastic model to determine the output sequence for cell death.

As discussed previously, the three sub-pathways are not necessarily equally expressed in all cells. The over- or under-activation of a sub-pathway in a particular cancerous cell is described by a probability, which indicates that each sub-pathway contributes to some extent to cell death. In this work, hence, cell death is determined by a weighted combination of the three sub-pathways. The respective weights of the three sub-pathways are assumed to be *p*_1_, *p*_2_ and *p*_3_ (*p*_1_ + *p*_2_ + *p*_3_ = 1). Due to the lack of prior knowledge, different combinations of weights are considered for the three sub-pathways and each combination of the weights (or probabilities) is referred to as a simulation strategy. Different strategies are implemented by the stochastic model in Fig. 6. The weights (as probabilities) are encoded into the non-Bernoulli sequences indicated by the combinations of control bits *C*_1_ and *C*_2_, respectively. As per the discussion in Stochastic Boolean network section, if the control bits of the multiplexer (MUX), i.e., *C*_1_ and *C*_2_, equal to ‘00’, then the output of sub-pathway 1 will be selected; if *C*_1_*C*_2_ = 01, then the output of sub-pathway 2 will be taken as the output; otherwise, cell survival is determined by the output of sub-pathway 3. Thus the output is affected by the assigned weights that reflect the impact of the three sub-pathways in cells.

## Results and Discussion

### Experimental and computational results

**Figure 7:**
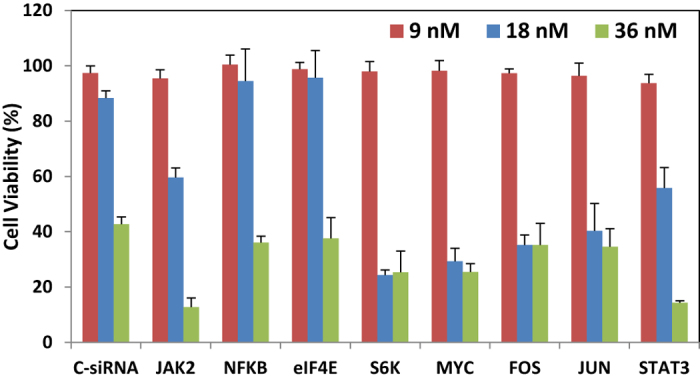
Experimental results for silencing individual targets. Cell viability after 9, 18 and 36 nM siRNA treatment was expressed as a percentage of un-treated cells (NT). Treatment with control (scrambled) siRNA (C-siRNA) was used to determine non-specific treatment toxicity, which was evident at 36 nM siRNA concentration, but not at lower concentrations.

The experimental results on silencing single targets are illustrated in Fig. 7. While treating cells at 9 nM siRNA concentration led to no significant changes in cell viability (i.e., drug concentration below the efficacy threshold), 18 and 36 nM siRNA treatments led to significant changes in cell viability. The 36 nM concentration for non-specific (scrambled) siRNA also gave some loss of cell viability, so that the results at this concentration were considered non-specific (i.e., due to general toxicity of the delivery system), thus only the results at 18 nM were considered for comparison to simulations. Based on experimental results, the effective gene nodes for suppression were JAK2 and Stat3 (partially effective), S6K, Myc, FOS, and JUN. There was minimal change in the cell viability by silencing NFkB and elF4E, as shown in Fig. 7.

**Figure 8:**
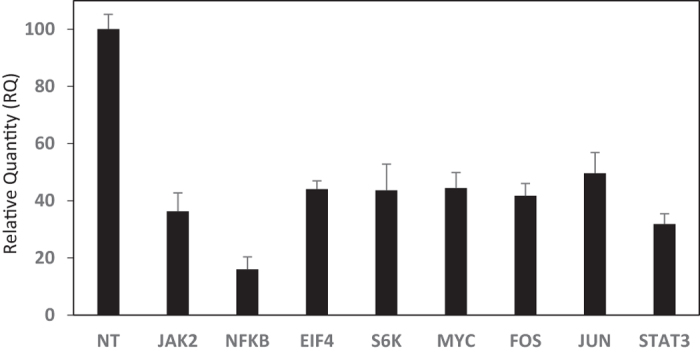
Relative quantity of targeted mRNAs after siRNA treatment (18 nm). The mRNA levels were determined after 72 hours of siRNA treatment and normalized with the level of mRNA in un-treated cells.

To confirm silencing of the targeted mRNA, the relative quantity of the specific mRNA after 18 nM siRNA treatment is shown in Fig. 8. Note that as compared to non-treated cells (NT), the mRNA levels of all targets were significantly reduced. An equivalent level of silencing was seen in all cases (~60%) although the NFkB silencing appeared to be slightly higher. The differences in obtained viabilities, therefore, seems to be dependent on the importance of each target physiologically, rather than the silencing efficiency obtained in the cell model.

In the stochastic simulation, different weights are assigned to the three sub-pathways. The weights of the three pathways are taken as the probabilities that each sub-pathway is dominant in the pathway. The probabilities are then encoded into the random binary bit streams for processing in stochastic computing. More specifically, the weights (or probabilities) are encoded into the select/control sequences to the multiplexers used in the stochastic computational models. Different sequences are generated for encoding the weights of the 16 strategies in the simulation. A single gene is selected from a set of candidate target nodes {JAK2, Stat3, NFkB, S6K, elF4E, JUN, FOS, Myc, and Mcl1}. If probabilistic drug delivery is considered, a probability of 0.6 is assigned for the effective function of the drug. This probability may vary for different genes or different drug doses. The drug delivery model in Fig. 4 is then applied to the selected candidate nodes. The obtained vulnerabilities by suppressing one of the genes {JAK2, Stat3, NFkB, S6K, elF4E, JUN, FOS, Myc, and Mcl1} are given in Table 1 for deterministic suppression and in Table 2 for probabilistic suppression.

**Figure 9:**
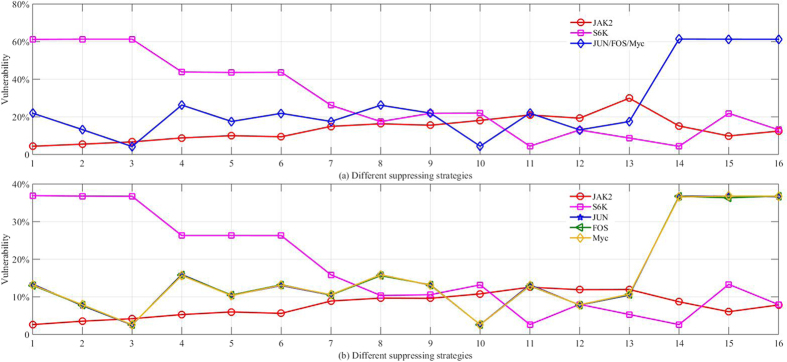
(**a**) Vulnerability of different gene nodes (JAK2, S6K, JUN, FOS, and Myc) for deterministic drug delivery. (**b**) Vulnerability of the different gene nodes for probabilistic drug delivery (drug effectiveness is assumed to be 60%). The horizontal axis indicated the different weight assignment strategy number as listed in Tables 1 and 2. The outcomes for JUN, FOS, and Myc overlap for deterministic silencing, but slightly vary for probabilistic drug delivery.

As revealed by the simulation results in Tables 1 and 2, suppressing genes NFkB and elF4E had no effect on survival, as indicated by a vulnerability value of 0 for these nodes. The JAK2, Stat3, S6K, JUN, FOS, Myc, and Mcl1 had a higher effect on the vulnerabilities. This is consistent with the experimental results in Fig. 7. For different weight assignment strategies where the activation of a particular pathway is altered, different genes act as the most effective target for suppression (i.e., resulting in different vulnerabilities). The outcomes from the significant nodes identified after deterministic and probabilistic gene silencing, are further illustrated in Fig. 9(a,b), respectively.

The relative effect of suppressing different genes on vulnerabilities is closely related to the weight assigned to each sub-pathway. The significance of Myc, for example, is most pronounced for scenarios #14 to #16, where the sub-pathway 3 was assigned a weight of 70%. The significance of S6K is most pronounced for scenarios #1 to #3, where the sub-pathway 2 was assigned a 70% weight. The vulnerabilities of probabilistic drug delivery were generally smaller than deterministic drug delivery for the same weight assignments, although the trends are similar in both approaches. For some targets, JUN, FOS, and Myc for example, the outcome on the vulnerabilities was similar irrespective of the simulation method employed.

**Figure 10:**
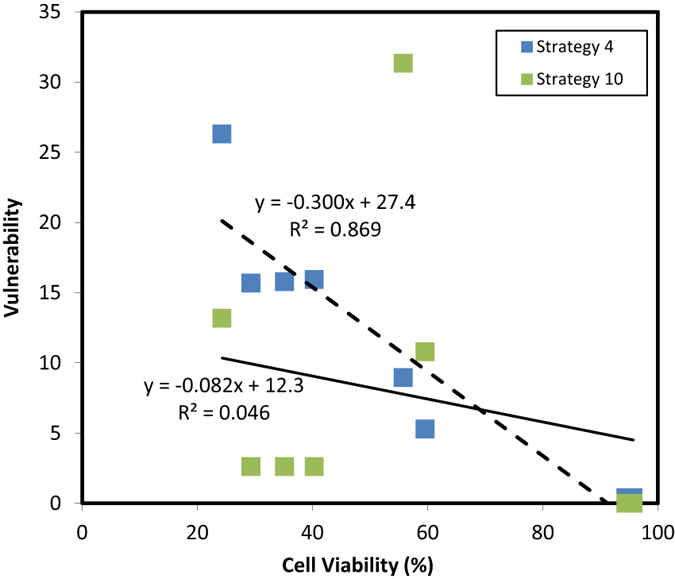
The correlations for strategy #4 (best fit) and strategy #10 (least fit).

The statistical analysis of the correlation between experimental results (cell viability from Fig. 7 at 18 nM) and computational results is presented in Table 3 for probabilistic drug delivery. For each strategy, a correlation between the cell viabilities for the 9 experimentally targeted genes and the corresponding vulnerabilities (from Table 2) was determined. Six out of the 16 strategies gave significant (*p* < 0.05) correlation between the experimental and simulation results (Table 3), where the best and least fits (based on *p* values) were obtained for strategy #4 and #10, respectively (Fig. 10). Best correlations were obtained for strategies #4, #5 and #6, where the sub-pathway 2 was assigned 50% weight percentage. Strategies #1, #8 and #15 also displayed significant correlations.

## Discussion

The fact that significant correlations were obtained for some strategies, but not all, was indicative of certain pathways of the developed network to be primarily active in the chosen cancer cell model. The modeling proposed in this study may allow one to quickly identify the vulnerabilities by altering the weight of each sub-pathway, before the time-consuming (and costly) experimental determination of vulnerabilities. If the vulnerabilities of a particular mediator are found to vary significantly depending on the weight of the sub-pathways, this will be indicative of a possible target that requires customization for therapy; i.e., it is likely that, when this target was employed for therapy, the response of tumors from different patients will be highly variable due to known heterogeneities in signaling pathways among the patients.

The targets whose vulnerabilities are not variable (i.e., as a function of the weights of sub-pathways) might be more universally acceptable targets for therapy, provided that their vulnerabilities are high. The fact that we have seen best correlations where the sub-pathway 2 was assigned the major weight fraction suggests that this pathway is particularly active in the chosen cell model.

In our network, NFkB and elF4E were found to be two targets whose vulnerabilities were nearly zero and should not make good candidates for targets. The current literature, however, provides some importance of NFkB in breast cancer survival; responses to some agents (e.g., 1*α*, 25-dihydroxyvitamin D3 and dexamethasone) activated NFkB signaling that led a pro-survival response in breast cancer cells^40^,^41^. The treatments in these studies involved broadly acting agents that regulate a multitude of pathways and the pro-survival effect might have been due to activation of mediators other than NFkB. Our approach was to specifically silence the intended target (NFkB), which indicated no apparent effect of NFkB on survival of breast cancer cells experimentally. This was consistent with the computational results. Nevertheless, it is likely that other mediators participate in the simulated network and these were not considered in our simulations. Future work will focus on improving the participants in the network to provide more comprehensive view of critical nodes as well as more accurately predict the experimental outcomes.

On the other hand, we found that Mcl1 was a target whose vulnerabilities were generally high irrespective of the weight of the pathway, indicating the possibility of being a good target for drug therapy. In fact, we have shown previously that the cell model used in this study was exceptionally sensitive to Mcl1 silencing^36^, and others reported similar data as well in other cell models of breast cancer^42^,^43^. Such targets with high vulnerabilities irrespective of the weight of each sub-pathway may be universal targets applicable to therapy of all breast cancer patients.

The computational results made predictions on the effective target genes that, if suppressed, have impact on the cell viability. These results are validated by the experimental results. Both indicate that the genes elF4E and NFkB had nearly no effect on the relative cell viability and that JAK2, Stat3, S6K, JUN, FOS, Myc, and Mcl1 are effective candidates to influence the value of the relative cell viability. This information is not only valuable in understanding the mechanisms involved in the enhanced proliferation and survival of cancer cells, but also could provide a tool to estimate the clinical outcome of targeting each of these proteins.

The observed effects in simulations are affected by the different weight assignment strategies, which are introduced to account for differential activation of different pathways in different types of breast cancer cells. A good correlation between the experimental and computational results was evident in certain simulation scenarios in the case of silencing single genes. Where a good correlation was obtained, it may confirm the activation of particular sub-pathways at chosen weight percentages. We anticipate clinical analysis on quantitative and mutational changes in target genes to guide weight assignments to different sub-pathways. While this assignment will rely on mutational analysis of patient samples, the model will provide more reliable data by taking into account the contributions of other sub-pathways that is not possible from simple mutational analysis on a particular target. This approach could have a significant impact on cancer treatment, where multiple intracellular pathways work in cooperation to support rapidly proliferating cells with enhanced survival potential and reliance on each of these pathways, and therefore, could create a versatile responsiveness to anticancer agents. We must note that the current work is performed with a single cell line and we anticipate that the response to siRNA therapy might vary if other breast cancer cell lines are used. Exploring this issue will be the basis of our future work. We expect that the significance of specific strategies (i.e., weight assessment of each sub-pathway) will vary for individual cell lines. This might be reminiscent of clinical situation, where the response to siRNA therapy might vary among the patient cells (i.e., the vulnerabilities of indivicual targets may vary). The proposed algorithm might be adjusted in this case to identify the significance of designated pathways.

## Additional Information

**How to cite this article**: Zhu, P. *et al*. Identification of Potential Drug Targets in Cancer Signaling Pathways using Stochastic Logical Models. *Sci. Rep.* **6**, 23078; doi: 10.1038/srep23078 (2016).

## Supplementary Material

Supplementary Information

## Figures and Tables

**Table 1:** Vulnerability of different nodes in the survival pathway (%) under deterministic drug delivery.

**Strategy**	**Sub-1**	**Sub-2**	**Sub-3**	**JAK2**	**Stat3**	**NFkB**	**S6K**	**elF4E**	**JUN**	**FOS**	**Myc**	**Mcl1**
#1	5%	70%	25%	4.35%	3.68%	0	61.20%	0	21.95%	21.95%	21.95%	25.63%
#2	15%	70%	15%	5.48%	11.12%	0	61.32%	0	13.21%	13.21%	13.21%	24.33%
#3	25%	70%	5%	6.67%	18.61%	0	61.32%	0	4.42%	4.42%	4.42%	23.03%
#4	20%	50%	30%	8.79%	14.90%	0	43.86%	0	26.26%	26.26%	26.26%	41.17%
#5	30%	50%	20%	9.98%	22.46%	0	43.63%	0	17.54%	17.54%	17.54%	40.00%
#6	25%	50%	25%	9.41%	18.77%	0	43.72%	0	21.86%	21.86%	21.86%	40.63%
#7	50%	30%	20%	14.93%	37.52%	0	26.14%	0	17.54%	17.54%	17.54%	55.06%
#8	50%	20%	30%	16.31%	37.65%	0	17.46%	0	26.17%	26.17%	26.17%	63.82%
#9	50%	25%	25%	15.53%	37.45%	0	21.88%	0	21.98%	21.98%	21.98%	59.44%
#10	70%	25%	5%	18.10%	52.44%	0	21.99%	0	4.39%	4.39%	4.39%	56.83%
#11	70%	5%	25%	20.99%	52.59%	0	4.38%	0	21.94%	21.94%	21.94%	74.53%
#12	70%	15%	15%	19.29%	52.53%	0	13.01%	0	13.06%	13.06%	13.06%	65.59%
#13	70%	10%	20%	29.92%	52.42%	0	8.72%	0	17.51%	17.51%	17.51%	69.93%
#14	25%	5%	70%	15.14%	18.81%	0	4.33%	0	61.36%	61.36%	61.36%	80.17%
#15	5%	25%	70%	9.83%	3.70%	0	21.86%	0	61.30%	61.30%	61.30%	65.00%
#16	15%	15%	70%	12.41%	11.25%	0	13.11%	0	61.28%	61.28%	61.28%	72.53%

Each sub-pathway was assigned a different weight percentage, as indicated.

The state of the targeted gene is suppressed to 0. Simulated sequence length is 500 k bits for the stochastic approach.

**Table 2:** Vulnerability of different nodes in the survival pathway (%) under probabilistic drug delivery.

**Strategy**	**Sub-1**	**Sub-2**	**Sub-3**	**JAK2**	**Stat3**	**NFkB**	**S6K**	**elF4E**	**JUN**	**FOS**	**Myc**	**Mcl1**
#1	5%	70%	25%	2.62%	2.27%	0	36.92%	0	13.37%	13.06%	13.15%	15.47%
#2	15%	70%	15%	3.52%	6.72%	0	36.78%	0	7.66%	7.78%	7.89%	14.67%
#3	25%	70%	5%	4.18%	11.08%	0	36.74%	0	2.53%	2.58%	2.59%	13.75%
#4	20%	50%	30%	5.28%	8.94%	0	26.29%	0	15.93%	15.77%	15.67%	24.57%
#5	30%	50%	20%	5.98%	13.56%	0	26.29%	0	10.40%	10.47%	10.34%	23.88%
#6	25%	50%	25%	5.60%	11.16%	0	26.28%	0	13.00%	13.20%	13.11%	24.15%
#7	50%	30%	20%	8.88%	22.49%	0	15.85%	0	10.47%	10.49%	10.57%	32.93%
#8	50%	20%	30%	9.66%	22.30%	0	10.35%	0	15.87%	15.63%	15.85%	38.12%
#9	50%	25%	25%	9.60%	22.72%	0	10.56%	0	13.09%	13.19%	13.12%	35.39%
#10	70%	25%	5%	10.79%	31.33%	0	13.18%	0	2.61%	2.60%	2.61%	34.01%
#11	70%	5%	25%	12.56%	31.57%	0	2.63%	0	13.19%	12.93%	12.98%	44.65%
#12	70%	15%	15%	11.89%	31.59%	0	8.00%	0	7.81%	7.85%	7.86%	39.51%
#13	70%	10%	20%	11.95%	31.49%	0	5.30%	0	10.47%	10.59%	10.70%	41.73%
#14	25%	5%	70%	8.68%	11.28%	0	2.61%	0	36.74%	36.72%	36.55%	48.05%
#15	5%	25%	70%	6.04%	2.26%	0	13.13%	0	36.80%	36.35%	36.80%	38.85%
#16	15%	15%	70%	7.84%	6.80%	0	7.94%	0	36.63%	36.79%	36.78%	43.60%

Each sub-pathway was assigned a different weight percentage, as indicated.

The state of the targeted gene is suppressed to 0, but the suppressing process occurs with a probability of *p* = 0.6. Simulated sequence length is 500 k bits for the stochastic approach.

**Table 3:** Significance of correlations between experimental and simulation results.

**No**	**1**	**2**	**3**	**4**	**5**	**6**	**7**	**8**
*r*^*2*^	0.593	0.442	0.255	0.869	0.672	0.815	0.477	0.548
p	0.025	0.072	0.202	0.001	0.013	0.002	0.058	0.036
**No**	**9**	**10**	**11**	**12**	**13**	**14**	**15**	**16**
*r*^2^	0.468	0.046	0.129	0.093	0.119	0.415	0.547	0.493
p	0.061	0.611	0.382	0.462	0.403	0.085	0.036	0.052

The *r*^2^ and p values are presented for different strategies listed in Table 2.
