# Supplementary material for: Identification of Potential Drug Targets in Cancer Signaling Pathways using Stochastic Logical Models
Source: Sci Rep. 2016 Mar 18;6:23078. doi: 10.1038/srep23078 (PMC4796823; doi:10.1038/srep23078)
Supplement: Supplementary Information [file srep23078-s1.pdf]

Identification of Potential Drug Targets in Cancer Signaling Pathway using Stochastic Logical Models.

Peican Zhu[1], Hamidreza Montazeri Aliabadi[2,3], Hasan Uludağ[3,*], Jie Han[1,*]

1 Department of Electrical and Computer Engineering, University of Alberta, Edmonton, AB T6G 2V4, Canada

2 School of Pharmacy, Chapman University, Irvine, CA 92618, USA

3 Department of Chemical and Material Engineering, University of Alberta, Edmonton, AB T6G 2V4, Canada

E-mail: hasan.uludag@ualberta.ca; jhan8@ualberta.ca

Supplementary file

Table S1: The sequences of the siRNAs used for this study.

Protein	Source		
JAK2	Qiagen (Cat. No.: S102659657)	Target Sequence	5’-AGCCATCATACGAGATCTTAA-3’
		Sense Strand	5’-CCAUCAUACGAGAUCUUAATT-3’
		Antisense Strand	5’-UUAAGAUCUCGUAUGAUGGCT-3’
NFκB	Qiagen (Cat. No.: S100300958)	Target Sequence	5’-AACAGAGAGGATTTCGTTTCC-3’
		Sense Strand	5’-CAGAGAGGAUUUCGUUUCCTT-3’
		Antisense Strand	5-GGAAACGAAAUCCUCUCUGTT-3’
elF4E	Qiagen (Cat. No.: S100300125)	Target Sequence	5’-AAGAGCGGCTCCACCACTAAA-3’
		Sense Strand	5’-GAGCGGCUCCACCACUAAATT-3’
		Antisense Strand	5’-UUUAGUGGUGGAGCCGCUCTT-3’
S6K	Qiagen (Cat. No.: S100301721)	Target Sequence	5’-GGGAGTTGGACCATATGAACT-3’
		Sense Strand	5’-GAGUUGGACCAUAUGAACUTT-3’
		Antisense Strand	5’-AGUUCAUAUGGUCCAACUCCC-3’
Myc	Qiagen (Cat. No.: S102662611)	Target Sequence	5’-CTCGGTGCAGCCGTATTTCTA-3’
		Sense Strand	5’-CGGUGCAGCCGUAUUUCUATT-3’
		Antisense Strand	5’-UAGAAAUACGGCUGCACCGAG-3’
FOS	Qiagen (Cat. No.: S102781429)	Target Sequence	5’-GACCAATATTATACTAAGAAA-3’
		Sense Strand	5’-CCAAUAUUAUACUAAGAAATT-3’
		Antisense Strand	5’-UUUCUUAGUAUAAUAUUGGTC-3’
JUN	Qiagen (Cat. No.: S100300580)	Target Sequence	5’-AAGAACGTGACAGATGAGCAG-3’
		Sense Strand	5’-GAACGUGACAGAUGAGCAGTT-3’
		Antisense Strand	5’-CUGCUCAUCUGUCACGUUCTT-3’
Stat3	Qiagen (Cat. No.: S102662338)	Target Sequence	5’-CAGCCTCTCTGCAGAATTCAA-3’
		Sense Strand	5’-GCCUCUCUGCAGAAUUCAATT-3’
		Antisense Strand	5’-UUGAAUUCUGCAGAGAGGCTG-3’

Table S2: The sequences of the primers used for qPCR analysis of selected proteins.

Protein of Interest		Sequence
β-Actin (endogenous protein)	Forward	5’-CCA CCC CAC TTC TCT CTA AGG A-3’
	Reverse	5’-AAT TTA CAC GAA AGC AAT GCT-3’
ACVR	Forward	5’-TGA GCA ATG GTA TAG TGG AGG ATT AC-3’
	Reverse	5’-CAG ACT ACC TTC CTC ATA TCT TCA AAA C-3’
JAK2	Forward	5’-AAC TGC AGA TGC ACA TCA TTA CCT-3’
	Reverse	5’-TCG AAA TTG GGC CAT GAC A-3’
NFκB	Forward	5’-AAT GGG CTA CAC CGA AGC AA-3’
	Reverse	5’-CAG CGA GTG GGC CTG AGA-3’
elF4E	Forward	5’-GAG GAC GAT GGC TAA TTA CAT TGA-3’
	Reverse	5’-GCA CAG AAG TGT CTC TAG CCA AAA-3’
S6K	Forward	5’-AAT CCG ATC ACC TCG AAG ATT TAT-3’
	Reverse	5’-CTG TGC TGG CCG AAG CA-3’
Myc	Forward	5’-TGG TCT TCC CCT ACC CTC TCA-3’
	Reverse	5’-AGA ATC CGA GGA CGG AGA GAA-3’
FOS	Forward	5’-CTG AAG ACC GAG CCC TTT GA-3’
	Reverse	5’-GGA GCG GGC TGT CTC AGA-3’
JUN	Forward	5’-GAG AGG AAG CGC ATG AGG AA-3’
	Reverse	5’-TCC AGC CGG GCG ATT-3’
Stat3	Forward	5’-ACA ACA TGT CAT TTG CTG AAA TCA-3’
	Reverse	5’-TCC TTG GGA ATG TCA GGA TAG AG-3’

Table S3 Logical relationships for multiple interactions with a single gene in sub-pathway 1. Standards of pathway representation in Figure 2(a) facilitate the understanding of the pathway (SBGN in [Ref. 44])

Biological relationship	Logical expression
(JAK, MUC1) → Stat3	Stat3 = OR(JAK, MUC1)
(Bcl-2,MCL1,Survivin) ⊣ Apoptosis	Apoptosis = OR(NOT(Bcl-2),NOT(MCL1),NOT(Survivin))

Table S4 Logical relationships for multiple interactions with a single gene in sub-pathway 2. Standards of pathway representation in Figure 2(b) facilitate the understanding of the pathway (SBGN in [Ref. 44])

Biological relationship	Logical expression
(MUC1,GFs) → Ras	Ras = OR(MUC1,GFs)
(Ras,GFs,FAK) ⊣ PI3K	PI3K = OR(Ras,GFs,FAK)
(Akt,S6K) ⊣ BAD	BAD = OR(NOT(Akt),NOT(S6K))
(S6K, NFkB, elF4E) → Bcl-2	Bcl-2 = OR(S6K,AND(NFkB,elF4E))
(S6K, NFkB, elF4E) → MCL1	MCL1 = OR(S6K,AND(NFkB,elF4E))
(S6K, NFkB, elF4E) → Survivin	Survivin = OR(S6K,AND(NFkB,elF4E))
(BAD, Ceramide) ⊣ (Bcl-2, MCL1, Survivin) → Apoptosis	Apoptosis = OR(BAD,AND(Ceramide, OR(NOT(Bcl-2),NOT(MCL1),NOT(Survivin))))

Table S5 Logical relationships for multiple interactions with a single gene in sub-pathway 3. Standards of pathway representation in Figure 2(c) facilitate the understanding of the pathway (SBGN in [Ref. 44])

Biological relationship	Logical expression
(MUC1,GFs) → Ras	Ras = OR(MUC1,GFs)
(JAK, Raf) → MEK	MEK = OR(JAK,Raf)
(MEKK,Src) → Myc	Myc = OR(MEKK,Src)
(JUN, FOS, Myc) → MCL1	MCL1 = AND(JUN,FOS,Myc)
(Myc, JUN) → Bcl-2	Bcl-2 = AND(Myc,JUN)
(Bcl-2, MCL1, Survivin) → Apoptosis	Apoptosis = OR(NOT(Bcl-2),NOT(MCL1),NOT(Survivin))
